# Fecal glycoprotein 2 is a marker of gut microbiota dysbiosis and systemic inflammation

**DOI:** 10.1186/s13099-024-00657-1

**Published:** 2024-10-19

**Authors:** Fabian Frost, Stefan Weiss, Johannes Hertel, Malte Rühlemann, Corinna Bang, Andre Franke, Matthias Nauck, Marcus Dörr, Henry Völzke, Dirk Roggenbuck, Peter Schierack, Uwe Völker, Georg Homuth, Ali A. Aghdassi, Matthias Sendler, Markus M. Lerch, Frank U. Weiss

**Affiliations:** 1 Department of Medicine A, University Medicine Greifswald, Ferdinand-Sauerbruch- Straße, 17475 Greifswald, Germany; 2 Department of Functional Genomics, Interfaculty Institute for Genetics and Functional Genomics, University Medicine Greifswald, Greifswald, Germany; 3 Department of Psychiatry and Psychotherapy, University Medicine Greifswald, Greifswald, Germany; 4 Institute of Clinical Molecular Biology, Kiel University, Kiel, Germany; 5 Institute of Clinical Chemistry and Laboratory Medicine, University Medicine Greifswald, Greifswald, Germany; 6 Department of Internal Medicine B, University Medicine Greifswald, Greifswald, Germany; 7 Institute for Community Medicine, University Medicine Greifswald, Greifswald, Germany; 8 Institute of Biotechnology, Faculty Environment and Natural Sciences, Brandenburg University of Technology Cottbus-Senftenberg, Senftenberg, Germany; 9 Faculty of Health Sciences Brandenburg, Brandenburg University of Technology Cottbus-Senftenberg, Senftenberg, Germany

**Keywords:** GP2, Exocrine, Pancreas, Pancreatitis, IBD, Biomarker

## Abstract

**Background:**

Antimicrobial autoantigenic glycoprotein 2 (GP2) is an important component of the innate immune system which originates from the exocrine pancreas as well as from the small intestines. The relationship of GP2 with the intestinal microbiome as well as the systemic implications of increased fecal GP2 levels are, however, still unclear. Therefore, fecal samples from 2,812 individuals of the Study of Health in Pomerania (SHIP) were collected to determine GP2 levels (enzyme-linked immunosorbent assay) and gut microbiota profiles (16 S rRNA gene sequencing). These data were correlated and associated with highly standardised and comprehensive phenotypic data of the study participants.

**Results:**

Fecal GP2 levels were increased in individuals with higher body mass index and smokers, whereas lower levels were found in case of preserved exocrine pancreatic function, female sex or a healthier diet. Moreover, higher GP2 levels were associated with increased serum levels of high-sensitivity C-reactive protein, loss of gut microbial diversity and an increase of potentially detrimental bacteria (*Streptococcus*, *Haemophilus*, *Clostridium XIVa*, or *Collinsella*). At the same time, predicted microbial pathways for the biosynthesis of beneficial short-chain fatty acids or lactic acid were depleted in individuals with high fecal GP2. Of note, GP2 exhibited a stronger association to overall microbiome variation than calprotectin.

**Conclusion:**

Fecal GP2 is a biomarker of gut microbiota dysbiosis and associated with increased systemic inflammation. The intestines may be more important as origin for GP2 than pancreatic acinar cells. Future studies need to investigate the potential clinical value in disease specific patient cohorts.

**Supplementary Information:**

The online version contains supplementary material available at 10.1186/s13099-024-00657-1.

## Background

Gut bacteria are not passive bystanders but carry out important metabolic functions and their interplay with antimicrobial components of the host’s immune system is crucial for the maintenance of health. Pathological alterations of the gut microbiome, so-called dysbiosis, have been implicated in the development and progression of various diseases [1]. Several factors that shape or affect the gut microbiome composition have been described so far, but many more variables influencing the gut microbiota variation remain unknown [2]. Recently, we identified the exocrine pancreas as one of the most important host factors which influence the gut microbiota composition in the general population [3, 4]. A preserved exocrine pancreatic function not only supports a healthy gut microbiome composition but also predicts increased microbiome stability in the future [5]. How these observations correlate in detail has not been completely understood. On the one hand, loss of exocrine pancreatic function may change the nutritional substrate composition in the large intestines and select for specific bacteria according to their primary energy source [3, 6]. On the other hand, secretory proteins from the pancreatic acinar cell, like microbiome-sensing pancreatic glycoprotein 2 (GP2), have been described to play an important role in the innate immune response against different commensal bacteria or opportunistic pathogens like *Escherichia (E.) coli* and the associated potential loss of tolerance [7–9]. Expression of GP2 has also been detected in duodenal glands, at the apical plasma membrane of small intestinal M cells and presumably colonic enteroendocrine L cells [7, 10, 11]. To date, four isoforms of the pancreatic secretory granule membrane major glycoprotein (GP2) have been described (PubMed accession numbers: NP_001007241.2, NP_001493.2, NP_001007242.2, and NP_001007243.2). In microfold (M) cells, GP2 acts as a microbiome-sensing receptor of the intestinal follicle-associated epithelium (FAE) which is involved in transcytosing FimH-positive bacteria and subsequently triggering an immune response against them [7] while soluble GP2 interacts with the bacterial adhesin FimH, reducing infection rates of enterotoxigenic *E. coli* in cell line models [9]. In inflammatory bowel disease and autoimmune liver disease, GP2 appears to be a potential autoantigenic target related to severity and tumourigenesis [12, 13]. The loss of tolerance to GP2 in form of GP2 autoantibodies appears to be a stratification factor of Crohns’ disease and seems related to changes of the gut microbiota composition [14–16]. Patients suffering from IBD show higher fecal GP2 levels, whereas GP2 deficient colitis mice exhibit a phenotype characterized by increased intestinal inflammation and epithelial attachment of *E. coli* [17]. This again indicates, that GP2 can prevent epithelial attachment and penetration of commensal *E. coli*. Therefore, it is assumed that GP2 is necessary as a regulator to prevent chronic intestinal inflammation [17]. Furthermore, in primary sclerosing cholangitis, an autoimmune liver disease associated with a specific change of the gut microbiota, the occurrence of GP2 IgA is a predictor of disease severity and cholangiocarcinogenesis [18, 19]. However, in the general population without manifest gastrointestinal disease, the role and importance of GP2 is much less clear. In the present study we determined fecal GP2 levels and microbiota profiles in 2,812 individuals of the Study of Health in Pomerania (SHIP) to identify phenotypic factors which associate with high fecal GP2 levels as well as to determine their correlation with changes in the gut microbiome composition.

## Methods

### Study population

All individuals were participants of the Study of Health in Pomerania (SHIP) which is a longitudinal population-based cohort study encompassing the two independent cohorts SHIP-TREND and SHIP-START [20]. Participants of both cohorts were randomly selected from the general population of North-east Germany, as SHIP aims to investigate the prevalence and characteristics of common diseases and their risk factors in this area [21]. Fecal samples were collected during the initial recruitment of SHIP-TREND (*n* = 4,420, 2008–2012) and during the second follow-up of the SHIP-START cohort (*n* = 2,333, 2008–2012, initial recruitment 1997–2001). GP2 measurements were available for a total of 2,919 individuals. Of these we removed 107 participants with GP2 levels outside the mean +/- 3 standard deviations (SD), a history of pancreatic disease or intake of antibiotics at the time of sample collection, leaving 2,812 datasets for analysis. Corresponding intestinal microbiome data based on 16 S rRNA gene sequencing was available for 2,671 individuals. All participants provided written informed consent and the study was approved by the local ethics committee of the University Medicine Greifswald (BB 39/08 and BB 122/13).

### Collection of fecal samples

The participants were provided two fecal collection tubes with and without DNA-stabilizing EDTA-buffer (0.5 M Tris, 10 mM NaCl, 100 mM EDTA, pH 7.0). Fecal samples were collected by the study participants as described before [3] at home and brought to the study center individually or shipped by mail.

### Determination of fecal glycoprotein 2 (GP2) levels

For determination of GP2 levels in stool, native fecal samples were analysed using the Pancreatitis GP2 ELISA (GA Generic Assays, Berlin, Germany) according to the manufacturer´s protocol. The assay detects the larger isoform of GP2 [22]. Briefly, 25 mg of stool were homogenized in 1.25 ml extraction buffer and centrifuged for 10 min at 3000 g. 20 µl of supernatant were subsequently analysed in 96-well plates, which included GP2 standards as well as positive and negative controls. Final optical density (OD) measurements were done in a spectrophotometer (SpectraMax 190) from Molecular Devices and GP2 values were calculated as [ng GP2/mg stool] from an internal GP2 standard curve.

### 16 S rRNA gene sequencing of fecal samples

Determination of gut microbiota profiles using 16 S rRNA gene sequencing was performed as described before in detail [3]. In brief, DNA was extracted from fecal samples stored in a DNA-stabilizing EDTA-buffer using the PSP Spin Stool DNA Kit (Stratec Biomedical AG, Birkenfeld, Germany) following the manufacturer’s instructions and isolates were stored at -20 °C degrees until sequencing of the V1/V2 region of the bacterial 16 S rRNA gene using the 27 F and the 338R primers on a MiSeq platform (Illumina, San Diego, USA).

### Taxonomy assignment and predicted metagenomics

CASAVA 1.8.2 (https://support.illumina.com/sequencing/sequencing_software/casava) was employed to create MiSeq FastQ files. Data amplicon processing was then performed using the open-source software DADA2 [23] according to the recommended procedure for large datasets (https://benjjneb.github.io/dada2/bigdata.html) adapted to the V1/V2 16 S rRNA gene region as described before [24]. For assignment of taxonomy, a Bayesian classifier and the Ribosomal Database Project (RDP) training set version 16 were used. All data were rarefied to 10,000 reads per sample before analysis. Metagenomic microbial functions were predicted based on the amplicon sequence variants (ASVs) derived from the DADA2 pipeline using the PICRUSt2 package [25] with the standardized workflow as described at https://github.com/picrust/picrust2/wiki/Workflow.

### Other laboratory and phenotypic data

Fecal pancreatic elastase levels were determined using a monospecific pancreatic elastase ELISA assay (BIOSERV Diagnostics GmbH, Germany) according to the manufacturer’s protocol as described before in detail [3]. Fecal calprotectin levels were determined using the RIDASCREEN Calprotectin assay (R-Biopharm, Darmstadt, Germany). Laboratory parameters (alanine aminotransferase (ALT), creatinine, high-density lipoprotein (HDL), high-sensitivity CRP (hs-CRP), low-density lipoprotein (LDL), and thyroid-stimulating hormone (TSH)) were measured on a Dimension VISTA platform (Siemens Healthcare Diagnostics, Eschborn Germany). To determine glycated haemoglobin concentrations (HbA1c) high-performance liquid chromatography (Bio-Rad Diamat, Munich, Germany) was performed. The CKD-EPI equation was employed for estimation of glomerular filtration rate (eGFR) [26]. Chronic kidney disease was assigned in case of eGFR values below 60 ml/min. Body mass index (BMI) was calculated as kilograms per square meter of the body height. Presence of diabetes mellitus was assigned in case of a positive patient history of diabetes in combination with current treatment (dietetically, orally, and/or insulin injections) or in case of HbA1c measurements ≥ 6.5% or random blood glucose levels ≥ 11.1 mmol/L. Hypothyroidism was assigned in case of thyroid hormone replacement therapy or an TSH ≥ 4 mU/L. Dyslipidemia was considered in case of LDL/HDL ratios > 3.5 or > 3 for males or females, respectively, or current anti-lipid medication. For estimation of the daily alcohol intake in grams alcohol per day all alcoholic beverages that had been consumed over the last 30 days were counted and its average alcoholic contend calculated. Smoking was considered if the participant was a present smoker. Fatty liver disease was diagnosed in case of hyperechogenic liver tissue using high resolution ultrasound (Vivid i, GE Healthcare, Chicago, Illinois, USA) in B mode in combination with serum alanine aminotransferase (ALT) levels belonging to the upper 25% of the investigated population. To assess the quality of diet, a food frequency score (FFS) based on consumption data from 15 food categories (meat, sausage, fish, boiled potatoes, pasta, rice, raw vegetables, boiled vegetables, fruits, whole grain/black/crisp bread, oats/cornflakes, eggs, cake/cookies, sweets and savoury snacks) was calculated as described elsewhere [24, 27, 28], where higher values indicated a healthier diet. Participants were classified as physically active if they reported performing at least two hours of sport per week regularly during winter and summer. Arterial hypertension was considered in case of antihypertensive medication intake or a systolic or diastolic blood pressure ≥ 140 or ≥ 90 mmHg, respectively. Manifest atherosclerotic disease was assigned in case of a history of myocardial infarction or stroke.

### Data analysis

All statistical analyses were implemented in ‘R’ (v. 3.6.3, https://www.R-project.org/) [29]. All figures were created using the packages ‘ggplot2’ or ‘ggraph’ [30]. Square root transformation of continuous phenotype variables was performed prior to all association analyses. To analyse the association patterns of fecal GP2-levels with other phenotypic variables a two-step approach was performed: i) Simple linear regression analyses (function ‘lm’, ‘stats’ package) were performed with GP2-levels as outcome and the respective phenotypic variable as explanatory variables. ii) All variables that were significantly associated with GP2-levels in simple linear regression were combined as predictors in a multiple linear regression model to validate the robustness of the associations. Microbial beta diversity was calculated using a Bray-Curtis dissimilarity (function ‘vegdist’, ‘vegan’ package [31]) based on gut microbiota counts. Principal coordinate analysis (PCoA) was then performed (function ‘cmdscale’, ‘vegan’ package) and the contribution to the ordination result of different phenotypic variables analysed using the ‘envfit’ function (‘vegan’ package) assessing the statistical significance by 1,000 permutations. To analyse the association of individual gut microbial taxa (outcome) with fecal GP2-levels (explanatory variable), two different models were calculated including the potential confounding factors pancreatic elastase, age, sex, BMI, FFS, smoking, cohort, and sequencing batch: i) Linear regression model with log-transformed continuous gut microbiota abundance data. Only taxa with presence in at least 10% of all samples were considered. Zeroes were treated as NA to avoid spurious results due to zero-inflation. Outliers ± 3 SD away from the mean were removed in each taxon. All taxa were normalized to enable comparable effect estimates. ii) Logistic regression model with binary gut microbiota data (absent vs. present). Only taxa with presence in at least 10% but not more than 80% of samples were considered. The alpha diversity scores ‘Chao1 estimator’ or ‘Shannon diversity index’ (H), and ‘Simpson diversity number’ (N2) were computed using the ‘vegan’ package (Chao1 estimator: function ‘estimateR’; H and N2: function ‘diversity’). To analyse the association of microbial alpha diversity (outcome) with fecal GP2 levels (explanatory variable) a linear regression model was used including the same possible confounding factors as for the taxon - trait associations analyses. To analyse the association of predicted microbial pathways for short-chain fatty acid (SCFA) or lactic acid biosynthesis (outcome) with fecal GP2 levels (explanatory variable), the pathway gene abundance data were removed from outliers (± 3 SD), square root transformed and normalized. Again, the same possible confounding factors as for the taxon - trait association analyses were included into the model. All p-values derived from taxon-trait or pathway-trait associations were corrected for multiple testing using the method of ‘Benjamini & Hochberg’ and thereafter called q-values. P- or q-values < 0.05 were considered significant.

## Results

### GP2 association analysis with phenotypic factors

The complete study cohort comprised 2,812 individuals with available fecal GP2 levels. Median GP2 levels were 1,118.7 ng/g stool (215.4–3,659.1, first - third quartile). The phenotypic characteristics of the cohort are given in Table 1. Simple linear regression analyses revealed significant positive associations of fecal GP2 levels with smoking (*p* < 0.001), BMI (*p* < 0.001), alcohol consumption (*p* = 0.001), hs-CRP (*p* = 0.002), and fatty liver disease (*p* < 0.005) whereas inverse associations were found with higher FFS values (*p* < 0.001), female sex (*p* < 0.001), fecal elastase (*p* < 0.001), age (*p* < 0.001), and chronic kidney disease (*p* = 0.047). No associations were found for hypothyroidism, or cardiometabolic disorders such as diabetes mellitus, dyslipidemia, hypertension, or manifest atherosclerotic disease. After including all of the aforementioned significantly associated variables into one multiple regression model inverse associations with fecal GP2 levels remained for fecal elastase (*p* < 0.001), age (*p* = 0.001), female sex (*p* = 0.019), and FFS (*p* = 0.023), whereas positive associations were confirmed for BMI (*p* < 0.001), smoking (*p* = 0.007) and hs-CRP (*p* = 0.042).

**Table 1:** Phenotype characteristics and regression analysis

	Distribution	Missing	Simple regression			Multiple regression		
		(%)	Estimate	SE	p-value	Estimate	SE	p-value
Fecal Glycoprotein 2 (GP2, ng/g stool)	1118.7 (215.4-3659.1)	0	NA	NA	NA	NA	NA	NA
Age (years)	56.0 (44.0–66.0)	0	-2.7	0.6	< 0.001	-2.5	0.8	0.001
Body mass index (kg/m²)	27.4 (24.7–30.7)	0.1	5.8	1.4	< 0.001	6	1.7	< 0.001
Fecal elastase (µg/g stool)	503.1 (360.5-606.6)	0.3	-0.7	0.1	< 0.001	-0.6	0.1	< 0.001
Alcohol consumption (g/d)	3.9 (1.0-10.9)	1.5	1.2	0.3	0.001	0.4	0.4	0.278
Food frequency score	15.0 (12.0–17.0)	0.2	-7.2	1.4	< 0.001	-3.5	1.6	0.025
High-sensitivity CRP (mg/L)	1.2 (0.7–2.6)	5.6	2.7	0.9	0.002	1.8	0.9	0.045
Female sex (%)	52.7	0	-6.2	1.2	< 0.001	-3.5	1.5	0.018
Smoking (%)	19.4	0.1	7.9	1.6	< 0.001	4.7	1.7	0.006
Physically active (%)	25.5	0.1	0.2	1.4	0.859	NA	NA	NA
Proton-pump-inhibitors (%)	9.3	0	-2.4	2.1	0.268	NA	NA	NA
Diabetes mellitus (%)	11.7	0.4	0.6	1.9	0.775	NA	NA	NA
Fatty liver disease (%)	17.1	1	4.7	1.7	0.005	0.4	1.8	0.805
Dyslipidemia (%)	35.6	0.2	-0.6	1.3	0.624	NA	NA	NA
Hypertension (%)	47.2	0.3	-1.0	1.2	0.439	NA	NA	NA
Manifest atherosclerotic disease (%)	3.7	0.1	-6.2	3.3	0.060	NA	NA	NA
Chronic kidney disease (%)	7.0	0.2	-4.9	2.4	0.047	-1.5	2.7	0.571

All values are rounded to one decimal place. Continuous variables are displayed as median (first-third quartile) and binary variables as percentages. CRP: C-reactive protein. ^**#**^: Data only available for the SHIP-TREND cohort

### Association of GP2 with gut microbial beta diversity

**Fig. 1:**
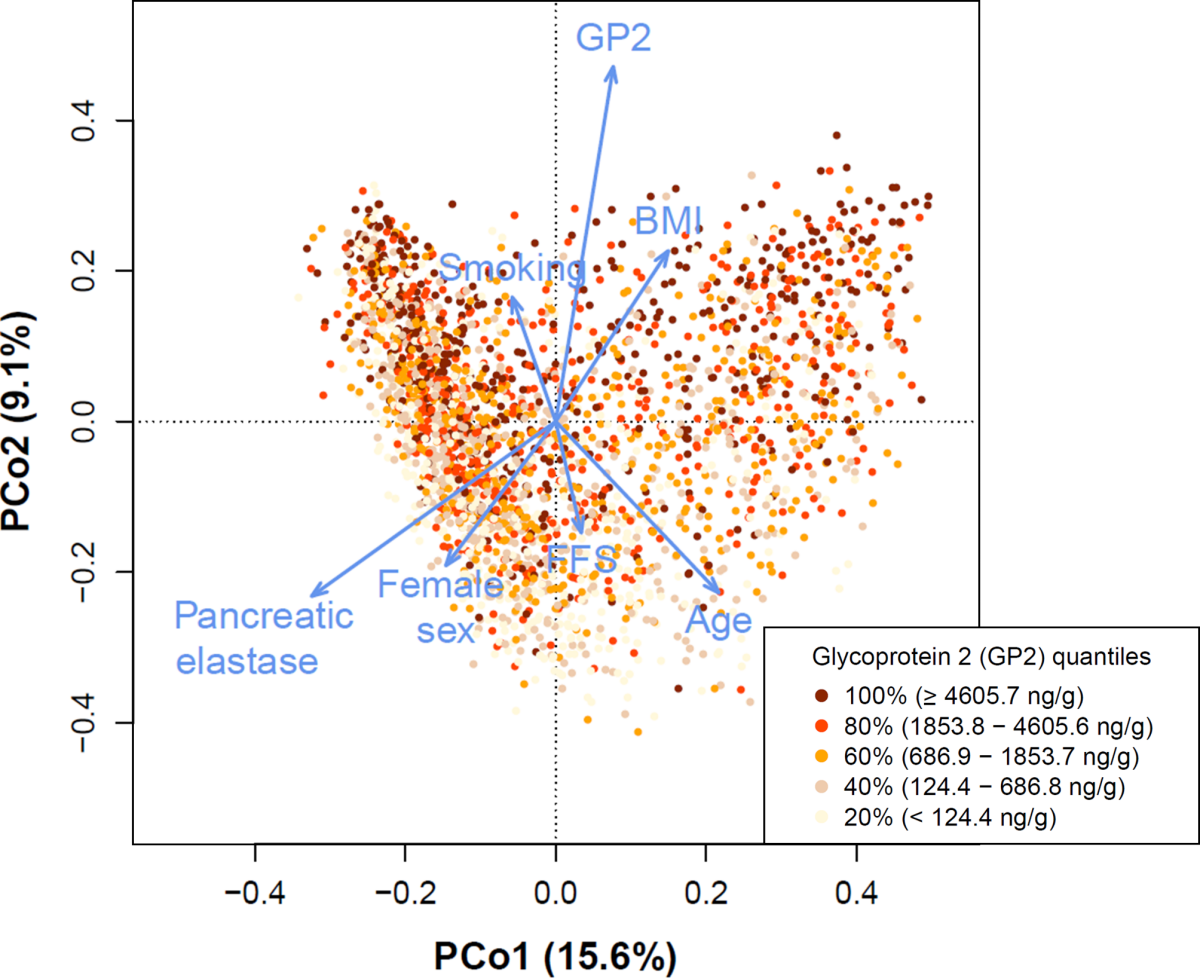
Contribution of GP2 levels and other important host factors to gut microbiota diversity. Shown are the two major PCo1 and PCo2 with each dot representing one individual gut microbiome sample, coloured according to the quantities of fecal GP2. The length of the blue arrows indicates the effect size of specific variable contributions to gut microbiota beta diversity. Variation in fecal GP2 and pancreatic elastase levels have the highest impact on microbial variation. BMI: Body mass index. FFS: Food frequency score (healthy diet)

The beta diversity metric ‘Bray-Curtis’ dissimilarity was calculated and a subsequent PCoA was performed to identify the microbial variation that can be attributed to different phenotype factors. We analysed only those factors that exhibited significant associations with GP2 levels in the multiple regression model. Figure 1 shows that the highest effect on microbial variation in this dataset could be attributed to changes in fecal GP2 (r²=13.5%, *p* < 0.001) or pancreatic elastase (r²=9.4%, *p* < 0.001). Significant associations were also detected with age (r²=5.8%, *p* < 0.001), BMI (r²=4.4%, *p* < 0.001), sex (r²=3.4%, *p* < 0.001), smoking (r²=1.8%, *p* < 0.001), or FFS (r²=1.4%, *p* < 0.001). Microbiome changes associated with higher GP2 levels were positively correlated with smoking or high BMI whereas an inverse association pattern was seen (along PCo2) for high pancreatic elastase levels, female sex, FFS (healthy diet), or age.

Next, we compared the effect sizes of the GP2-microbiome and calprotectin-microbiome associations. Calprotectin is an established non-specific biomarker for detection of intestinal inflammation [32]. For a total of 2,470 individuals, combined microbiome, GP2 and calprotectin data were available. In this subset, differences in calprotectin levels accounted for a much smaller proportion of the microbial variation (r²=1.7%, *p* < 0.001) when compared to GP2 (r²=10.8%, *p* < 0.001).

### Association patterns of GP2 with gut microbiota

**Fig. 2:**
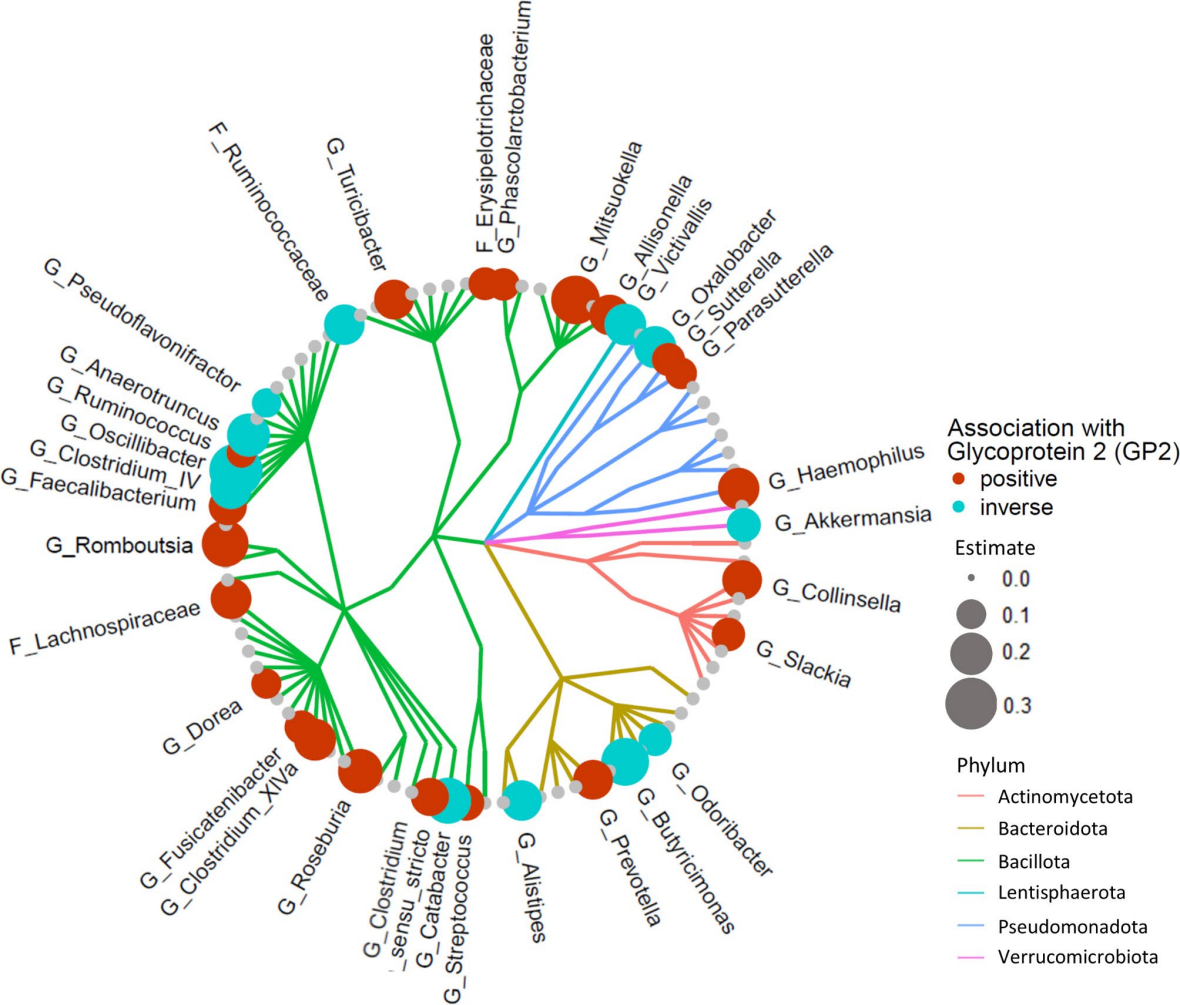
Association of GP2 levels with gut microbiota. Shown is a cladogram depicting microbial genera or families (continuous data) and their association with fecal GP2 levels. Significant results (q < 0.05) are depicted by a red (positive association) or blue dot (inverse association). The dot diameter corresponds to the magnitude of the regression effect estimate. Different phyla are colour coded. The analyses revealed marked gut microbiota changes in association to variation in fecal GP2 levels. G: Genus. F: (unclassified) Family

**Fig. 3:**
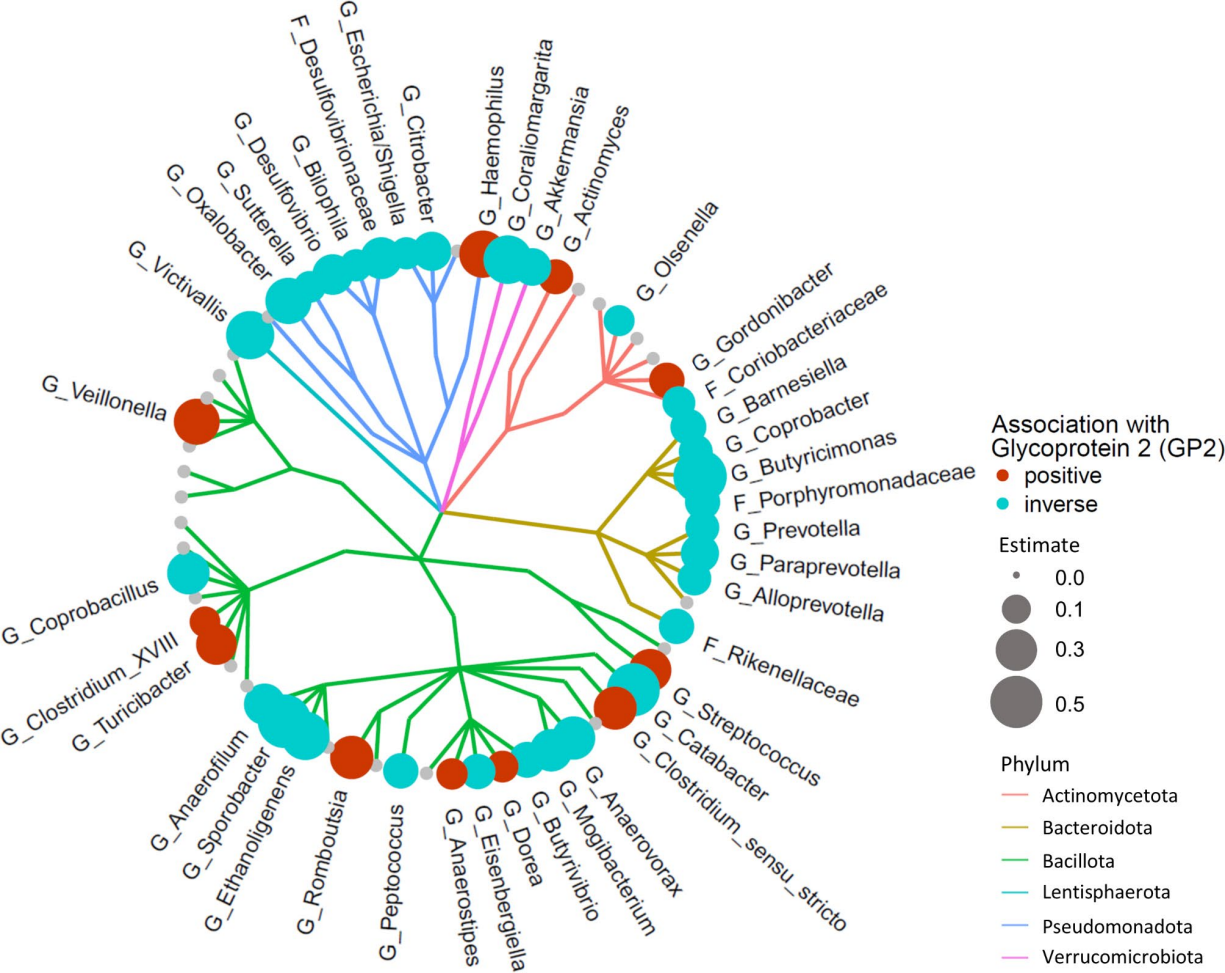
Association of GP2 levels with gut microbial presence-absence patterns. Shown is a cladogram depicting microbial genera or families (presence-absence data) and their association with fecal glycoprotein 2 (GP2) levels. Significant results (q < 0.05) are depicted by a red (positive association) or blue dot (inverse association). The dot diameter corresponds to the magnitude of the regression effect estimate. Different phyla are colour coded. The analyses revealed a loss of distinct microbial taxa in individuals with higher fecal GP2 levels. G: Genus. F: (unclassified) Family

A linear regression model was employed to analyse the association of fecal GP2 levels with individual gut microbial taxa. Frequencies of individual taxa are shown in Table S1. Significant association with increased GP2 levels were found for 21 taxa in this model whereas 12 taxa exhibited an inverse association (Fig. 2 and Table S2). A logistic regression model was used to analyse how fecal GP levels affect presence-absence patterns of gut microbiota and revealed 11 positive and 30 inverse associations (Fig. 3 and Table S3).

### Association patterns of GP2 with microbial alpha diversity

**Fig. 4:**
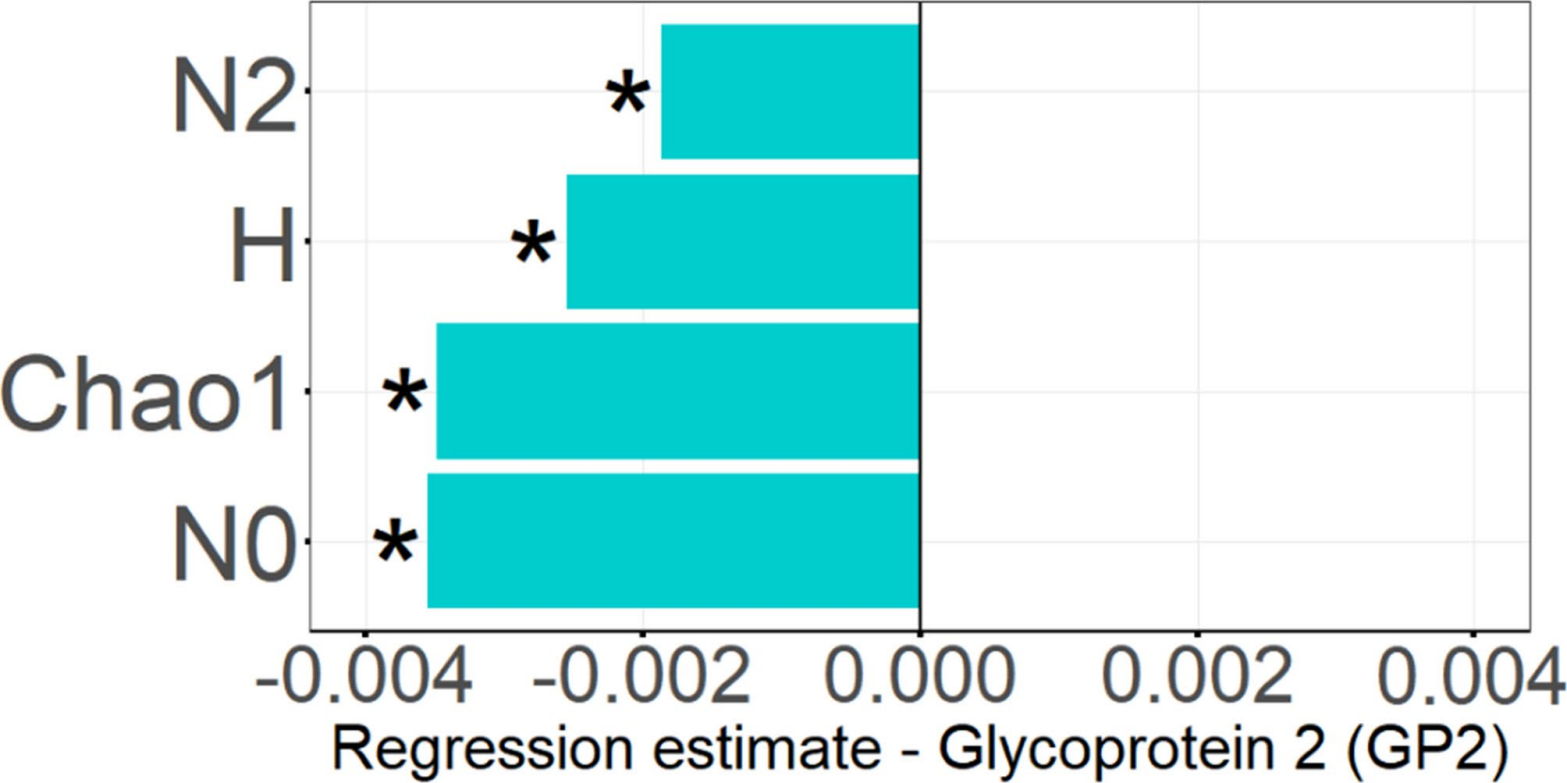
Association of GP2 levels with microbial alpha diversity reduction. The barplot shows inverse associations between fecal GP2 levels and the microbial diversity scores Simpson diversity number (N2), Shannon diversity index (H), Chao1 estimator and species richness (N0). *: indicates significant results (*p* < 0.05)

Higher GP2 levels were largely associated with the absence, but not the presence of different microbial taxa. To investigate the consequences of this finding on “in sample” microbial diversity (alpha diversity), the association of GP2 levels with the four different microbial diversity scores Simpson diversity number (N2), Shannon diversity index (H), Chao1 estimator, and species richness (N0) were calculated using a linear regression model. This analysis revealed a strong inverse association of higher GP2 levels with N2 (*p* = 0.002), H (*p* < 0.001), Chao1 (*p* < 0.001), and N0 (*p* < 0.001), (Fig. 4).

### Intestinal SCFA and lactic acid biosynthesis capability and its relation to GP2

**Fig. 5:**
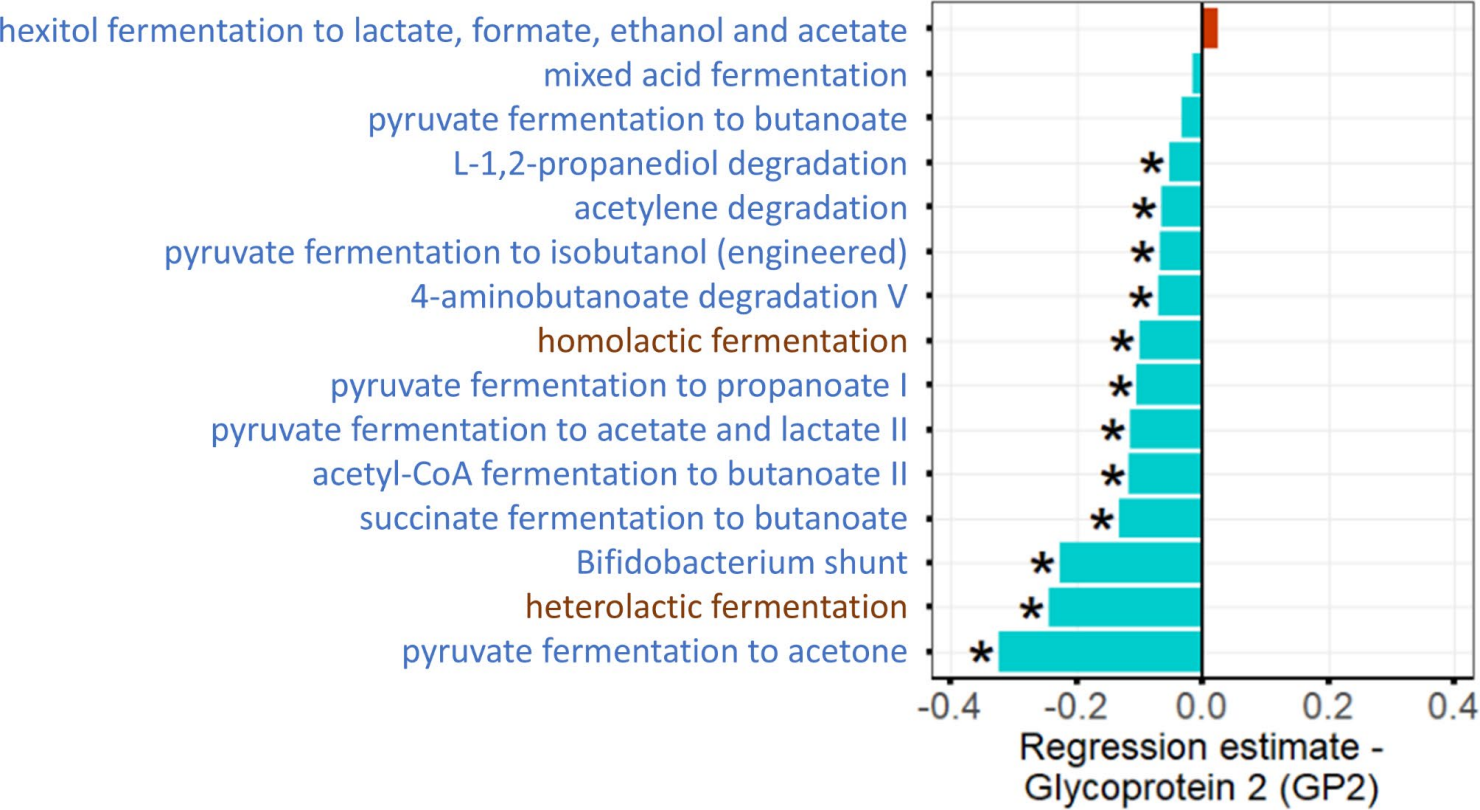
Association of GP2 levels with reduced SCFA and lactic acid biosynthesis capability. The barplot shows inverse (blue) or positive (red) associations between fecal GP2 levels and predicted microbial pathways for SCFA or lactic acid biosynthesis. Pathways that produce SCFA as primary or byproduct are coloured in blue. The analysis revealed a distinct reduction in the microbial capacity for SCFA or lactic acid biosynthesis in individuals with higher fecal GP2 levels. *: indicates significant results (q < 0.05)

Diversity changes that were found to correlate with high or low fecal GP2 levels included microbial taxa known to be involved in the biosynthesis of SCFA such as *Ruminococcaceae*, *Faecalibacterium*, *Lachnospiraceae*, or *Roseburia*. To investigate whether fecal GP2 levels are related with lower or higher SCFA or lactic acid biosynthesis capability, we analysed the predicted metagenomic pathways (PICRUSt) for the respective metabolites (details see methods). Figure 5 and Table S4 show that in individuals with higher fecal GP2 levels 12 out of 15 analysed predicted microbial pathways for SCFA or lactic acid biosynthesis were depleted.

## Discussion

In the present study, we analysed in a study sample of 2,812 individuals the relation of fecal GP2 levels to a range of different host factors, disease phenotypes, and the composition of the gut microbiome. It is supposed that the majority of fecal GP2 is secreted from zymogen granules of pancreatic acinar cells [33]. However, other sources such as Brunner’s glands in the duodenum, follicle-associated epithelium of small intestinal Peyer’s patches and colonic L cells have also been reported [7, 10, 11]. Elevated levels of GP2 have been found in both the exocrine pancreas and the intestinal mucosa in association with inflammatory processes in patients with Crohn’s disease or Crohn’s-like inflammation [8, 10, 17]. This finding may result from a tumor necrosis factor (TNF)-triggered GP2 synthesis and cause elevated secretion from the exocrine pancreas [17]. Alternatively, it could indicate GP2 secretion from intestinal sources into the gut lumen as we observed in our study cohort an inverse correlation of fecal GP2 levels with fecal pancreatic elastase levels. Fecal elastase is a clinical marker with higher values indicating a preserved exocrine pancreatic function. As GP2 is supposed to be secreted along with elastase and other zymogens from the secretory compartment of pancreatic acinar cells, this inverse correlation suggests an additional source of fecal GP2 other than the pancreas under such conditions. A putative candidate could be the duodenal exocrine Brunner’s glands demonstrating a higher synthesis and probably secretion of GP2 in patients with Crohn’s and celiac disease [11]. In our study, we also found an inverse correlation of fecal GP2 with a balanced diet (FFS) and female sex. On the other hand, high fecal GP2 levels were associated with smoking, higher BMI and increased systemic inflammation as indicated by high hs-CRP levels. The latter would support the assumption that inflammatory processes leading to raised TNF levels could trigger an elevated pancreatic secretion of GP2. No significant association was found with cardiovascular disease or diabetes mellitus. Regression analysis revealed a significant association with fatty liver disease, which was, however, lost after the inclusion of BMI as a stronger independent variable into the multiple regression analysis. Given the predictive power of mucosal loss of tolerance to particularly the larger GP2 isoform with disease severity in primary sclerosing cholangitis (PSC) [13], an autoimmune liver disease with male preponderance and dysbiosis [19], and the link of the male gender with higher fecal GP2 levels in this study, this may indicate a putative predisposition for the development of autoimmune cholangitis in males with high fecal GP2. In contrast, females develop more frequently primary biliary cholangitis, another autoimmune variant of cholangitis, with a different autoantigenic profile encompassing in particular mitochondrial targets [34]. Moreover, in another study PSC patients showed a marked reduction in a broad range of SCFA-producing bacteria similar to our observation of reduced predicted SCFA producing microbial pathways in association with higher GP2 levels [35]. Altogether, high fecal GP2 levels apparently indicate an unhealthy metabolic phenotype with increased systemic inflammation.

We further explored the underlying mechanisms leading to this observation and found that higher fecal GP2 levels were associated with gut microbiome changes, and that altered fecal GP2 levels explained a large part of the microbial variation in a PCoA. In linear and binary regression models, we detected 33 and 41 taxa, respectively, with significant associations with fecal GP2. Positive associations were found with *Clostridium XIVa*,* Collinsella* or the opportunistic pathogens *Haemophilus* and *Streptococcus*. In a recent publication we have shown that the presence of *Clostridium XIVa* in the gut microbiome of (still) healthy individuals associates with the development of fatty liver disease or diabetes mellitus over 5 years with a possible causal involvement [5]. Detection of *Collinsella* has been linked to diabetes mellitus, obesity and precedes the development of fatty liver disease [5, 36, 37]. Interestingly, we observed a reduced presence of the opportunistic Gram-negative pathogens *Escherichia/Shigella* and *Citrobacter*, in individuals with higher fecal GP2 levels. This observation may be explained by the fact that GP2 is an intestinal M-cells transcytotic receptor specific of type-I-piliated bacteria which participates in the mucosal immune response towards these bacteria [7].

In individuals with higher GP2 levels also some taxa which are known to be involved in SCFA biosynthesis, such as *Ruminococcaceae*, *Butyrivibrio*, *Faecalibacterium*, *Lachnospiraceae*, or *Roseburia*, showed altered abundance or presence. Analyses of predicted metagenomic microbial pathways revealed that the vast majority of these SCFA pathways including those necessary for lactic acid biosynthesis were depleted. SCFA such as acetate, propionate, or butyrate play important roles in the physiology of the intestinal tract. They represent an important energy source for colonic epithelia, have anti-inflammatory properties and promote the gut barrier function [38, 39]. Likewise, lactic acid producing bacteria have a beneficial effect on the gut barrier function [40], and the depletion of these microbial pathways may result in increased intestinal permeability, bacterial translocation, and increased (subclinical) systemic inflammation as indicated by higher serum hs-CRP levels. Even though our cross-sectional study did not detect associations of cardiovascular or manifest metabolic disease with higher fecal GP2-levels, elevated systemic inflammation may still increase the risk for cardiovascular disease or negatively affect metabolic conditions such as fatty liver disease or diabetes mellitus in the long-term [41, 42].

A further detrimental aspect of the gut microbiome in individuals with higher GP2 levels was a marked reduction of microbial alpha diversity. Low microbial diversity generally accompanies obesity [43, 44], gastrointestinal disorders such as chronic pancreatitis [45, 46] or inflammatory bowel disease [47, 48]. The consequence of low microbiota diversity is an increased instability of the gut microbiome which may facilitate the loss of potentially beneficial and the accumulation of pathogenic bacteria over time [5].

The strengths of the present study are the large sample size and the profound phenotypic characterization of the SHIP study participants. However, as this is a cross-sectional study it is not possible to prove the causality of the mechanisms involved. Whether elevated GP2 levels are the cause or consequence of the intestinal dysbiosis needs to be established in future studies which will have to involve long-term analysis of changes in GP2 and the microbiome. Unclear remains also the inverse correlation of GP2-levels and age. Whereas older individuals exhibited lower GP2-levels they are expected to have a higher risk of developing gastrointestinal disorders, potentially causing dysbiosis. An age-dependent reduced GP2 secretion capacity could be a possible explanation, however, the exact mechanism remains elusive and warrants further studies. Another limitation of the present study is that we did not have data available about probiotic intake or gut transit time, both of which may influence the gut microbiome composition [49]. Additionally, this study was performed in a geographically limited area and replication in other regions is needed.

## Conclusions

Our data indicate that the secretion of GP2 from the intestinal mucosa is another important source of fecal GP2 in addition to the secretion from pancreatic acinar cells. Fecal GP2 is a biomarker of gut microbiota dysbiosis associated with increased systemic inflammation. Of note, in this population-based sample the effect size of the association of GP2 with microbiome variation was distinctly higher than that of calprotectin, an established clinical biomarker of gastrointestinal inflammation [32]. The potential clinical value of GP2 as a biomarker of gut microbial dysbiosis and intestinal inflammation or its predictive potential of other systemic conditions needs to be clarified in future studies. Specifically, its potential value in detecting or monitoring inflammation in intestinal infections or inflammatory bowel disease will require investigations including disease specific patient cohorts and long-term follow-up assessments.

## Electronic supplementary material

Below is the link to the electronic supplementary material.

Supplementary Material 1

## Data Availability

The microbiome data is available online in the Zenodo repository (https://zenodo.org/records/11203667, DOI: 10.5281/zenodo.11203666). All phenotype data were obtained from the SHIP data management unit and can be applied for online through a data access application form (https://transfer.ship-med.uni-greifswald.de/FAIRequest/?lang=en).

## References

[1] Fan Y, Pedersen O. Gut microbiota in human metabolic health and disease. Nat Rev Microbiol. 2021;19(1):55–71. 32887946 10.1038/s41579-020-0433-9

[2] Zhernakova A, Kurilshikov A, Bonder MJ, Tigchelaar EF, Schirmer M, Vatanen T, et al. Population-based metagenomics analysis reveals markers for gut microbiome composition and diversity. Science. 2016;352(6285):565–9. 27126040 10.1126/science.aad3369 PMC5240844

[3] Frost F, Kacprowski T, Rühlemann M, Bülow R, Kühn J-P, Franke A, et al. Impaired exocrine pancreatic function associates with changes in intestinal microbiota composition and diversity. Gastroenterology. 2019;156(4):1010–5. 30391469 10.1053/j.gastro.2018.10.047

[4] Pietzner M, Budde K, Rühlemann M, Völzke H, Homuth G, Weiss FU, et al. Exocrine pancreatic function modulates plasma metabolites through changes in gut microbiota composition. J Clin Endocrinol Metab. 2021;106(5):e2290–8. 33462612 10.1210/clinem/dgaa961 PMC8186556

[5] Frost F, Kacprowski T, Rühlemann M, Pietzner M, Bang C, Franke A, et al. Long-term instability of the intestinal microbiome is associated with metabolic liver disease, low microbiota diversity, diabetes mellitus and impaired exocrine pancreatic function. Gut. 2021;70(3):522–30. 33168600 10.1136/gutjnl-2020-322753 PMC7873430

[6] Ritz S, Hahn D, Wami HT, Tegelkamp K, Dobrindt U, Schnekenburger J. Gut microbiome as a response marker for pancreatic enzyme replacement therapy in a porcine model of exocrine pancreas insufficiency. Microb Cell Fact. 2020;19(1):221. 33272255 10.1186/s12934-020-01482-2 PMC7713139

[7] Hase K, Kawano K, Nochi T, Pontes GS, Fukuda S, Ebisawa M, et al. Uptake through glycoprotein 2 of FimH(+) bacteria by M cells initiates mucosal immune response. Nature. 2009;462(7270):226–30. 19907495 10.1038/nature08529

[8] Werner L, Paclik D, Fritz C, Reinhold D, Roggenbuck D, Sturm A. Identification of pancreatic glycoprotein 2 as an endogenous immunomodulator of innate and adaptive immune responses. J Immunol. 2012;189(6):2774–83. 22891285 10.4049/jimmunol.1103190

[9] Bartlitz C, Kolenda R, Chilimoniuk J, Grzymajło K, Rödiger S, Bauerfeind R, et al. Adhesion of Enteropathogenic, Enterotoxigenic, and Commensal *Escherichia coli* to the Major Zymogen Granule Membrane Glycoprotein 2. Appl Environ Microbiol. 2022;88(5):e0227921. 35020452 10.1128/aem.02279-21 PMC8904060

[10] Derer S, Brethack A-K, Pietsch C, Jendrek ST, Nitzsche T, Bokemeyer A, et al. Inflammatory bowel disease-associated GP2 autoantibodies inhibit mucosal Immune response to adherent-invasive Bacteria. Inflamm Bowel Dis. 2020;26(12):1856–68. 32304568 10.1093/ibd/izaa069

[11] Roggenbuck D, Goihl A, Sowa M, Lopens S, Rödiger S, Schierack P, et al. Human glycoprotein-2 expressed in Brunner glands - a putative autoimmune target and link between Crohn’s and coeliac disease. Clin Immunol. 2023;247:109214. 36608744 10.1016/j.clim.2022.109214

[12] Roggenbuck D, Hausdorf G, Martinez-Gamboa L, Reinhold D, Büttner T, Jungblut PR, et al. Identification of GP2, the major zymogen granule membrane glycoprotein, as the autoantigen of pancreatic antibodies in Crohn’s disease. Gut. 2009;58(12):1620–8. 19549613 10.1136/gut.2008.162495

[13] Wunsch E, Norman GL, Milkiewicz M, Krawczyk M, Bentow C, Shums Z, et al. Anti-glycoprotein 2 (anti-GP2) IgA and anti-neutrophil cytoplasmic antibodies to serine proteinase 3 (PR3-ANCA): antibodies to predict severe disease, poor survival and cholangiocarcinoma in primary sclerosing cholangitis. Aliment Pharmacol Ther. 2021;53(2):302–13. 33159471 10.1111/apt.16153 PMC7821312

[14] Degenhardt F, Dirmeier A, Lopez R, Lang S, Kunst C, Roggenbuck D, et al. Serologic Anti-GP2 antibodies are Associated with genetic polymorphisms, fibrostenosis, and need for Surgical Resection in Crohn’s Disease. Inflamm Bowel Dis. 2016;22(11):2648–57. 27753692 10.1097/MIB.0000000000000936 PMC5082182

[15] Roggenbuck D, Reinhold D, Baumgart DC, Schierack P, Conrad K, Laass MW. Autoimmunity in Crohn’s Disease-A putative stratification factor of the clinical phenotype. Adv Clin Chem. 2016;77:77–101. 27717419 10.1016/bs.acc.2016.06.002

[16] Cummings D, Cruise M, Lopez R, Roggenbuck D, Jairath V, Wang Y, et al. Loss of tolerance to glycoprotein 2 isoforms 1 and 4 is associated with Crohn’s disease of the pouch. Aliment Pharmacol Ther. 2018;48(11–12):1251–9. 30411391 10.1111/apt.15034

[17] Kurashima Y, Kigoshi T, Murasaki S, Arai F, Shimada K, Seki N, et al. Pancreatic glycoprotein 2 is a first line of defense for mucosal protection in intestinal inflammation. Nat Commun. 2021;12(1):1067. 33594081 10.1038/s41467-021-21277-2 PMC7887276

[18] Jendrek ST, Gotthardt D, Nitzsche T, Widmann L, Korf T, Michaels MA, et al. Anti-GP2 IgA autoantibodies are associated with poor survival and cholangiocarcinoma in primary sclerosing cholangitis. Gut. 2017;66(1):137–44. 27406039 10.1136/gutjnl-2016-311739

[19] Sabino J, Vieira-Silva S, Machiels K, Joossens M, Falony G, Ballet V, et al. Primary sclerosing cholangitis is characterised by intestinal dysbiosis independent from IBD. Gut. 2016;65(10):1681–9. 27207975 10.1136/gutjnl-2015-311004 PMC5036217

[20] Völzke H, Schössow J, Schmidt CO, Jürgens C, Richter A, Werner A et al. Cohort Profile Update: the study of Health in Pomerania (SHIP). Int J Epidemiol 2022;51(6):372–383. 10.1093/ije/dyac034 35348705

[21] Völzke H, Alte D, Schmidt CO, Radke D, Lorbeer R, Friedrich N, et al. Cohort profile: the study of health in Pomerania. Int J Epidemiol. 2011;40(2):294–307. 20167617 10.1093/ije/dyp394

[22] Roggenbuck D, Goihl A, Hanack K, Holzlöhner P, Hentschel C, Veiczi M, et al. Serological diagnosis and prognosis of severe acute pancreatitis by analysis of serum glycoprotein 2. Clin Chem Lab Med. 2017;55(6):854–64. 27837595 10.1515/cclm-2016-0797

[23] Callahan BJ, McMurdie PJ, Rosen MJ, Han AW, Johnson AJA, Holmes SP. DADA2: high-resolution sample inference from Illumina amplicon data. Nat Methods. 2016;13(7):581–3. 27214047 10.1038/nmeth.3869 PMC4927377

[24] Frost F, Kacprowski T, Rühlemann M, Weiss S, Bang C, Franke A, et al. Carrying asymptomatic gallstones is not associated with changes in intestinal microbiota composition and diversity but cholecystectomy with significant dysbiosis. Sci Rep. 2021;11(1):6677. 33758296 10.1038/s41598-021-86247-6 PMC7988160

[25] Douglas GM, Maffei VJ, Zaneveld JR, Yurgel SN, Brown JR, Taylor CM, et al. PICRUSt2 for prediction of metagenome functions. Nat Biotechnol. 2020;38(6):685–8. 32483366 10.1038/s41587-020-0548-6 PMC7365738

[26] Levey AS, Stevens LA, Schmid CH, Zhang YL, Castro AF, Feldman HI, et al. A new equation to estimate glomerular filtration rate. Ann Intern Med. 2009;150(9):604–12. 19414839 10.7326/0003-4819-150-9-200905050-00006 PMC2763564

[27] Luedemann J, Schminke U, Berger K, Piek M, Willich SN, Döring A, et al. Association between behavior-dependent cardiovascular risk factors and asymptomatic carotid atherosclerosis in a general population. Stroke. 2002;33(12):2929–35. 12468793 10.1161/01.str.0000038422.57919.7f

[28] Winkler G, Döring A. Validation of a short qualitative food frequency list used in several German large scale surveys. Z Ernahrungswiss. 1998;37(3):234–41. 9800314 10.1007/pl00007377

[29] R Core Team. R: A language and environment for statistical computing. R Foundation for Statistical Computing. https://www.R-project.org/ 2020.

[30] Wickham H. Ggplot2: Elegrant graphics for data analysis / Hadley Wickham; with contributions by Carson Sievert. Second edition. Switzerland: Springer; 2016. (Use R!).

[31] Oksanen J, Blanchet FG, Friendly M, Kindt R, Legendre P, McGlinn D et al. vegan: Community Ecology Package. R package version 2.5-6. https://CRAN.R-project.org/package=vegan 2019.

[32] Jukic A, Bakiri L, Wagner EF, Tilg H, Adolph TE. Calprotectin: from biomarker to biological function. Gut. 2021;70(10):1978–88. 34145045 10.1136/gutjnl-2021-324855 PMC8458070

[33] Hoops TC, Rindler MJ. Isolation of the cDNA encoding glycoprotein-2 (GP-2), the major zymogen granule membrane protein. Homology to uromodulin/Tamm-Horsfall protein. J Biol Chem. 1991;266(7):4257–63. 1999417

[34] Coppel RL, McNeilage LJ, Surh CD, van de Water J, Spithill TW, Whittingham S, et al. Primary structure of the human M2 mitochondrial autoantigen of primary biliary cirrhosis: dihydrolipoamide acetyltransferase. Proc Natl Acad Sci U S A. 1988;85(19):7317–21. 3174635 10.1073/pnas.85.19.7317 PMC282177

[35] Kummen M, Thingholm LB, Rühlemann MC, Holm K, Hansen SH, Moitinho-Silva L, et al. Altered gut microbial metabolism of essential nutrients in primary sclerosing Cholangitis. Gastroenterology. 2021;160(5):1784–e17980. 33387530 10.1053/j.gastro.2020.12.058 PMC7611822

[36] Frost F, Storck LJ, Kacprowski T, Gärtner S, Rühlemann M, Bang C, et al. A structured weight loss program increases gut microbiota phylogenetic diversity and reduces levels of *Collinsella* in obese type 2 diabetics: a pilot study. PLoS ONE. 2019;14(7):e0219489. 31318902 10.1371/journal.pone.0219489 PMC6638920

[37] Lambeth SM, Carson T, Lowe J, Ramaraj T, Leff JW, Luo L, et al. Composition, diversity and abundance of gut microbiome in Prediabetes and Type 2 diabetes. J Diabetes Obes. 2015;2(3):1–7. 26756039 10.15436/2376-0949.15.031 PMC4705851

[38] Parada Venegas D, La Fuente MK, Landskron G, González MJ, Quera R, Dijkstra G, et al. Short chain fatty acids (SCFAs)-Mediated gut epithelial and Immune Regulation and its relevance for inflammatory Bowel diseases. Front Immunol. 2019;10:277. 30915065 10.3389/fimmu.2019.00277 PMC6421268

[39] Smith PM, Howitt MR, Panikov N, Michaud M, Gallini CA, Bohlooly-Y M, et al. The microbial metabolites, short-chain fatty acids, regulate colonic Treg cell homeostasis. Science. 2013;341(6145):569–73. 23828891 10.1126/science.1241165 PMC3807819

[40] Ren C, Dokter-Fokkens J, Figueroa Lozano S, Zhang Q, de Haan BJ, Zhang H, et al. Lactic acid Bacteria may impact intestinal barrier function by modulating Goblet cells. Mol Nutr Food Res. 2018;62(6):e1700572. 29333697 10.1002/mnfr.201700572 PMC5900975

[41] Tsalamandris S, Antonopoulos AS, Oikonomou E, Papamikroulis G-A, Vogiatzi G, Papaioannou S, et al. The role of inflammation in diabetes: current concepts and future perspectives. Eur Cardiol. 2019;14(1):50–9. 31131037 10.15420/ecr.2018.33.1 PMC6523054

[42] Yousuf O, Mohanty BD, Martin SS, Joshi PH, Blaha MJ, Nasir K, et al. High-sensitivity C-reactive protein and cardiovascular disease: a resolute belief or an elusive link? J Am Coll Cardiol. 2013;62(5):397–408. 23727085 10.1016/j.jacc.2013.05.016

[43] Le Chatelier E, Nielsen T, Qin J, Prifti E, Hildebrand F, Falony G, et al. Richness of human gut microbiome correlates with metabolic markers. Nature. 2013;500(7464):541–6. 23985870 10.1038/nature12506

[44] Turnbaugh PJ, Hamady M, Yatsunenko T, Cantarel BL, Duncan A, Ley RE, et al. A core gut microbiome in obese and lean twins. Nature. 2009;457(7228):480–4. 19043404 10.1038/nature07540 PMC2677729

[45] Frost F, Weiss FU, Sendler M, Kacprowski T, Rühlemann M, Bang C, et al. The gut microbiome in patients with chronic pancreatitis is characterized by significant dysbiosis and overgrowth by opportunistic pathogens. Clin Transl Gastroenterol. 2020;11(9):e00232. 33094959 10.14309/ctg.0000000000000232 PMC7494146

[46] Jandhyala SM, Madhulika A, Deepika G, Rao GV, Reddy DN, Subramanyam C, et al. Altered intestinal microbiota in patients with chronic pancreatitis: implications in diabetes and metabolic abnormalities. Sci Rep. 2017;7:43640. 28255158 10.1038/srep43640 PMC5334648

[47] Manichanh C, Rigottier-Gois L, Bonnaud E, Gloux K, Pelletier E, Frangeul L, et al. Reduced diversity of faecal microbiota in Crohn’s disease revealed by a metagenomic approach. Gut. 2006;55(2):205–11. 16188921 10.1136/gut.2005.073817 PMC1856500

[48] Zuo T, Ng SC. The gut microbiota in the Pathogenesis and therapeutics of inflammatory bowel disease. Front Microbiol. 2018;9:2247. 30319571 10.3389/fmicb.2018.02247 PMC6167487

[49] Procházková N, Falony G, Dragsted LO, Licht TR, Raes J, Roager HM. Advancing human gut microbiota research by considering gut transit time. Gut. 2022;72(1):180–91. 36171079 10.1136/gutjnl-2022-328166 PMC9763197

[50] Weiss S, Frost F. SHIP gut microbiome dataset for GP2 analysis; 2024.

